# Potential role of circulating long noncoding RNA MALAT1 in predicting disease risk, severity, and patients' survival in sepsis

**DOI:** 10.1002/jcla.22968

**Published:** 2019-07-13

**Authors:** Feng Geng, Wei Liu, Li Yu

**Affiliations:** ^1^ Department of ICU, The Central Hospital Of Wuhan, Tongji Medical College Huazhong University of Science and Technology Wuhan China

**Keywords:** lncRNA MALAT1, miR‐125b, prognosis, risk, sepsis, severity

## Abstract

**Background:**

This study aimed to investigate the plasma long noncoding RNA metastasis‐associated lung adenocarcinoma transcript 1 (lncRNA MALAT1) expression with risk, severity, inflammation level, and prognosis in sepsis.

**Methods:**

One hundred and ninety sepsis patients and 190 health controls (HCs) were consecutively recruited. Blood samples within 24 hours after admission of sepsis patients and those on enrollment of HCs were collected, and then, plasma was separated for lncRNA MALAT1 and miR‐125b expressions detections by RT‐qPCR. In sepsis patients, clinical data and 28‐day mortality were recorded, and plasma inflammatory cytokines expressions were detected by ELISA.

**Results:**

Plasma lncRNA MALAT1 expression was elevated in sepsis patients than HCs (*P* < 0.001), and ROC curve disclosed that it had a good value in predicting sepsis risk with an area under curve (AUC) of 0.823 (95% CI: 0.783‐0.864). Additionally, lncRNA MALAT1 expression was positively correlated with Scr (*P* = 0.005), WBC (*P* = 0.017), CRP (*P* < 0.001), PCT (*P* < 0.001), TNF‐α (*P* < 0.001), IL‐8 (*P* < 0.001), IL‐17 (*P* = 0.001), APACHE II score (*P* < 0.001), and SOFA score (*P* < 0.001). LncRNA MALA1 expression was elevated in deaths compared with survivors (*P* < 0.001) and could predict the risk of 28‐day mortality with an AUC of 0.755 (95% CI: 0.682‐0.828). Accumulating survival was worse in patients with lncRNA MALAT1 high expression compared with patients who had lncRNA MALAT1 low expression (*P* < 0.001). Moreover, lncRNA MALAT1 expression was negatively correlated with miR‐125b level in both sepsis patients (*P* < 0.001) and HCs (*P* < 0.001).

**Conclusion:**

LncRNA MALAT1 could be developed as a potential biomarker for facilitating diagnosis and management in sepsis patients.

## 1 INTRODUCTION

Sepsis, a systemic inflammatory response syndrome (SIRS) triggered by imbalanced and intricate host response to infection, attacks over 19 million persons annually and is responsible for approximately 30% of mortality in the intensive care unit (ICU).^1^, ^2^ Multiple risk factors for sepsis have been identified through years of investigations, such as very old age, immunosuppressive diseases, and cancers, which assist in the prevention of sepsis to some extent, and along with the reduced time delay to initial intervention, the survival of sepsis patients has been improved over the past decades.^3^, ^4^, ^5^ However, despite a decrease in in‐hospital mortality, the sepsis survivors still experience disability, chronic health problems, and readmission to hospital.^6^, ^7^, ^8^, ^9^ Thus, the prevention, diagnosis, and personalized management of sepsis remain to be optimized.

Long noncoding RNAs (lncRNAs) are a category of promising noncoding RNAs that have been found to interplay with multiple factors in various diseases, which are characterized by a length more than 200 nt and almost no protein‐coding function.^10^, ^11^, ^12^ However, although lncRNAs are not involved in protein coding, these molecules are able to mediate gene levels at different aspects, including transcription, post‐transcription processing, and so on.^13^ LncRNA metastasis‐associated lung adenocarcinoma transcript 1 (MALAT1) locates on chromosome 11 has been found to be essential in the regulation of tumorigenesis and inflammation, and for instance, it is reported that lncRNA MALAT1 promotes inflammation‐related hepatocellular carcinoma by binding to the chromatin‐remodeling subunit BRG1.^14^ Although it seems that lncRNA MALAT1 plays a dual role in inflammation according to the previous findings, recent studies report that lncRNA MALAT1 might be a pro‐inflammatory gene in sepsis.^15^, ^16^, ^17^ Therefore, we presumed that lncRNA MALAT1 may have the potential to serve as a biomarker for risk prediction of sepsis and prognosis in patients with sepsis. Nonetheless, the diagnostic and prognostic role of lncRNA MALAT1 in sepsis patients remains largely unknown.

Thus, the aim of this study was to investigate the plasma lncRNA MALAT1 expression with risk, severity, inflammation level, and prognosis in sepsis.

## 2 MATERIALS AND METHODS

### 2.1 Participants

This study consecutively recruited 190 sepsis patients who were admitted to intensive care units (ICUs) of The Central Hospital of Wuhan Hospital from January 2015 to December 2018. The patients included in this study were diagnosed as sepsis according to the Surviving Sepsis Campaign: International Guidelines for Management of Severe Sepsis and Septic Shock, 2012.^4^ Patients were excluded if they were less than 18 years old, accompanied with primary malignancies, received immunosuppressant before enrollment, were pregnant, lactating, human immunodeficiency virus (HIV)‐positive, or in end‐of‐life conditions. In addition, 190 healthy subjects who underwent physical examination in The Central Hospital of Wuhan were enrolled as healthy controls (HCs), and all of them had no history of severe infection or malignancies, or other obvious abnormalities by physical examination. The study protocol was approved by Ethics Committee of The Central Hospital of Wuhan. All participants or their guardians signed informed consents.

### 2.2 Data collection

Patients’ clinical data were collected, which consisted of age, gender, body mass index (BMI) smoke, history of chronic obstructive pulmonary disease (COPD), history of cardiomyopathy, history of chronic kidney failure, history of cirrhosis, serum creatinine (Scr), albumin, white blood cell (WBC), C‐reactive protein (CRP), procalcitonin (PCT), acute physiology and chronic health evaluation (APACHE) II score, and sequential organ failure assessment (SOFA) score. The APACHE II score and SOFA score were assessed within 24 hours after ICU admission. After hospital admission, all enrolled patients were followed up to 28 days, and 28‐day mortality was calculated, and according to that, the accumulating survival was calculated as well.

### 2.3 Samples collection and measurements

Blood samples of sepsis patients were collected in anticoagulant tube within 24 hours after ICU admission, and blood samples of HCs were collected in anticoagulant tube on the enrollment. All the blood samples were processed immediately after collection for the separation of plasma. The conditions were as follows: The whole blood samples were centrifuged at 1600 g for 10 minutes at 4°C, and supernatant was transferred to the Eppendorf (EP) tube, then were centrifuged again at 2500 g for 10 minutes at 4°C. After centrifugation, the plasma was collected and stored at −80°C until determination. LncRNA MALAT1 and miR‐125b relative expressions in plasma were determined using real‐time quantitative polymerase chain reaction (RT‐qPCR). The levels of inflammatory cytokines including tumor necrosis factor‐α (TNF‐α), interleukin‐1β (IL‐1β), IL‐6, and IL‐17 in plasma were determined by enzyme‐linked immunosorbent assay (ELISA) using commercial human ELISA Kits (eBioscience) following the manufacturer's protocol.

### 2.4 RT‐qPCR

First, the total RNA was elicited from plasma by using the TRIzol reagent (Invitrogen) and then was assessed by spectrometer for its purity. Then, the total RNA was reversely transcribed to complementary DNAs by PrimeScript RT reagent Kit (Takara). Subsequently, qPCR was performed by using TB Green Fast qPCR Mix (Takara), and the relative expressions of lncRNA and miRNA were calculated according to the 2^−ΔΔt^ formula using GAPDH as internal reference for lncRNA MALAT1 and U6 as internal reference for miR‐125b. In addition, the primers used in the present study were as follows: lncRNA MALAT1, forward: TCCTAAGGTCAAGAGAAGTGTCAG, reverse: GTGGCGATGTGGCAGAGAA; GAPDH, forward: GAGTCCACTGGCGTCTTCAC, reverse: ATCTTGAGGCTGTTGTCATACTTCT; U6, forward: CTCGCTTCGGCAGCACA, reverse: AACGCTTCACGAATTTGCGT; miR‐125b, forward: ACACTCCAGCTGGGTCCCTGAGACCCTAACTT, reverse: TGTCGTGGAGTCGGCAATTC.

### 2.5 Statistical analysis

Statistical analysis was performed using SPSS 24.0 (IBM), figure was plotted using GraphPad Prism 7.00 (GraphPad Software). Data were presented as mean ± standard deviation (SD), median (interquartile range, IQR), or count (percentage). Comparisons were determined by Wilcoxon rank sum test. Correlations were determined by Spearman's rank correlation test. Receiver operating characteristic (ROC) curves were plotted, and the area under curve (AUC) with 95% confidence interval (CI) was calculated to assess the discrimination ability of LncRNA MALAT1 and miR‐125b relative expressions between sepsis patients and HCs or between deaths and survivors. Kaplan‐Meier curve was used to illustrate accumulating survival, and accumulating survival difference between groups was determined by log‐rank test. All tests were 2‐sided, and *P* value < 0.05 was considered as significant.

## 3 RESULTS

### 3.1 Clinical characteristics of sepsis patients

There were 127 males and 63 females in sepsis patients with a mean age of 58.5 ± 13.8 years and a mean BMI of 23.1 ± 5.5 kg/m^2^ (Table 1). The numbers of patients who had history of COPD, cardiomyopathy, chronic kidney failure, and cirrhosis were, respectively, 33 (17.4%), 70 (36.8%), 14 (7.4%), and 35 (18.4%). And the mean values of APACHE II score and SOFA score were 12.9 ± 5.7 and 5.1 ± 2.8, respectively. In addition, the medians of TNF‐α, IL‐6, IL‐8, and IL‐17 were 174.1 (103.7‐272.0) pg/mL, 63.1 (35.8‐129.9) pg/mL, 52.7 (23.8‐131.8) pg/mL, and 123.5 (61.6‐183.5) pg/mL.

**Table 1:** Clinical characteristics of sepsis patients

Items	Sepsis patients (N = 190)
Age (y), mean ± SD	58.5 ± 13.8
Gender (male/female)	127/63
BMI (kg/m^2^), mean ± SD	23.1 ± 5.5
Smoke, No. (%)	58 (30.5)
History of COPD, No. (%)	33 (17.4)
History of cardiomyopathy, No. (%)	70 (36.8)
History of chronic kidney failure, No. (%)	14 (7.4)
History of cirrhosis, No. (%)	35 (18.4)
Scr (mg/dL), median (IQR)	1.6 (1.1‐2.4)
Albumin (g/L), median (IQR)	25.6 (21.3‐33.0)
WBC (×10^9^/L), median (IQR)	18.6 (3.2‐28.2)
CRP (mg/L), median (IQR)	98.7 (52.3‐150.0)
PCT (ng/mL), median (IQR)	12.6 (7.5‐24.4)
APACHE II score, mean ± SD	12.9 ± 5.7
SOFA score, mean ± SD	5.1 ± 2.8
TNF‐α (pg/mL), median (IQR)	174.1 (103.7‐272.0)
IL‐6 (pg/mL), median (IQR)	63.1 (35.8‐129.9)
IL‐8 (pg/mL), median (IQR)	52.7 (23.8‐131.8)
IL‐17 (pg/mL), median (IQR)	123.5 (61.6‐183.5)

Abbreviations: APACHE, acute physiology and chronic health evaluation; BMI, body mass index; COPD, chronic obstructive pulmonary disease; CRP, C‐reactive protein; IL, interleukin; IQR, interquartile range; PCT, procalcitonin; Scr, serum creatinine; SD, standard deviation; SOFA, sequential organ failure assessment; TNF, tumor necrosis factor; WBC, white blood cell.

### 3.2 Predictive value of plasma lncRNA MALAT1 for sepsis risk

**Figure 1:**
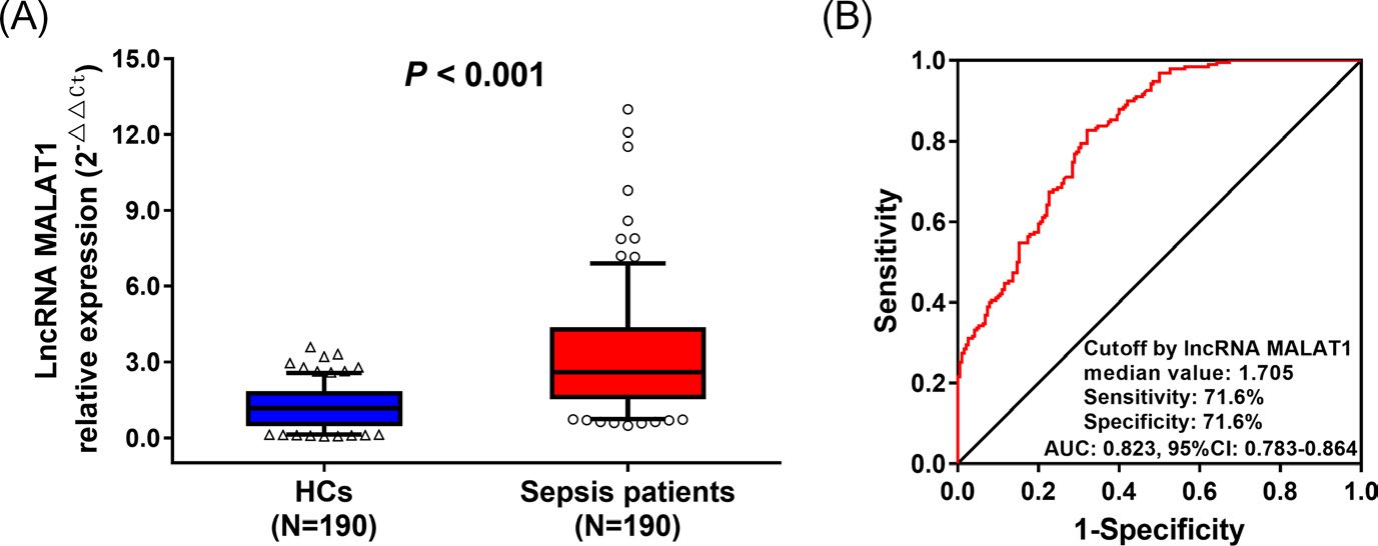
Expression of lncRNA MALAT1 in predicting sepsis risk. The plasma expression of lncRNA MALAT1 in sepsis patients and HCs (A), and its predictive value for sepsis risk by ROC curve analysis (B). Comparisons were determined by Wilcoxon rank sum test. ROC curves were plotted, and AUC with 95% confidence interval was calculated to assess the discrimination ability of LncRNA MALAT1 for sepsis risk. *P* value < 0.05 was considered significant. MALAT1, metastasis‐associated lung adenocarcinoma transcript 1; HCs, healthy controls; ROC, receiver operating characteristic; AUC, area under the curve

Plasma lncRNA MALAT1 expression was elevated in sepsis patients than that in HCs (*P* < 0.001; Figure 1A), and ROC curve analysis disclosed that lncRNA MALAT1 expression had a good value in predicting sepsis risk with an AUC of 0.823 (95%CI: 0.783‐0.864; Figure 1B). This result indicated that lncRNA MALAT1 expression in plasma may have potential in predicting sepsis risk.

### 3.3 Correlation of plasma lncRNA MALAT1 with clinical characteristics in sepsis patients

Regarding all the continuous variables of clinical characteristics in sepsis patients, lncRNA MALAT1 expression in plasma was positively correlated with Scr (*P* = 0.005), WBC (*P* = 0.017), CRP (*P* < 0.001), PCT (*P* < 0.001), TNF‐α (*P* < 0.001), IL‐8 (*P* < 0.001), and IL‐17 (*P* = 0.001) levels as well as the APACHE II score (*P* < 0.001) and SOFA score (*P* < 0.001; Table 2). However, lncRNA MALAT1 expression was not correlated with age (*P* = 0.643), BMI (*P* = 0.569), albumin (*P* = 0.099), or IL‐6 (*P* = 0.064) levels. As to the categorical variables of clinical characteristics, lncRNA MALAT1 level was not associated with gender (*P* = 0.519), smoke (*P* = 0.289), history of COPD (*P* = 0.739), history of cardiomyopathy (*P* = 0.402), history of chronic kidney failure (*P* = 0.127), or history of cirrhosis (*P* = 0.261; Table 3). These results suggested that lncRNA MALAT1 in plasma positively associated with severity and inflammation level in sepsis patients.

**Table 2:** Correlations of lncRNA MALAT1 relative expression with clinical characteristics (continuous variables)

Items	LncRNA MALAT1	
	*P* value	Correlation coefficient (*r*)
Age	0.643	−0.034
BMI	0.569	0.042
Scr	0.005	0.202
Albumin	0.099	−0.120
WBC	0.017	0.173
CRP	<0.001	0.296
PCT	<0.001	0.280
APACHE II score	<0.001	0.430
SOFA score	<0.001	0.475
TNF‐α	<0.001	0.354
IL‐6	0.064	0.135
IL‐8	<0.001	0.331
IL‐17	0.001	0.236

Note

Correlation analyses were determined by Spearman's rank correlation test.

Abbreviations: APACHE, acute physiology and chronic health evaluation; BMI, body mass index; CRP, C‐reactive protein; IL, interleukin; LncRNA, long noncoding RNA; PCT, procalcitonin; Scr, serum creatinine; SOFA, sequential organ failure assessment; TNF, tumor necrosis factor; WBC, white blood cell.

**Table 3:** Correlations of lncRNA MALAT1 relative expression with clinical characteristics (categorical variables)

Items	LncRNA MALAT1, median (IQR)	*P* value
Gender		
Male	2.550 (1.618‐4.232)	0.519
Female	2.800 (1.606‐4.352)	
Smoke		
Yes	2.382 (1.686‐4.367)	0.289
No	2.709 (1.347‐4.185)	
History of COPD		
Yes	2.143 (1.649‐4.803)	0.739
No	2.693 (1.590‐4.227)	
History of cardiomyopathy		
Yes	2.450 (1.617‐4.152)	0.402
No	2.676 (1.585‐4.628)	
History of chronic kidney failure		
Yes	4.155 (1.889‐5.915)	0.127
No	2.554 (1.600‐4.230)	
History of cirrhosis		
Yes	1.852 (1.050‐4.412)	0.261
No	2.672 (1.663‐4.280)	

Note

Correlation analyses were determined by Wilcoxon rank sum test.

Abbreviations: COPD, chronic obstructive pulmonary disease; LncRNA, long noncoding RNA.

### 3.4 Predictive value of plasma lncRNA MALAT1 for 28‐day mortality in sepsis patients

**Figure 2:**
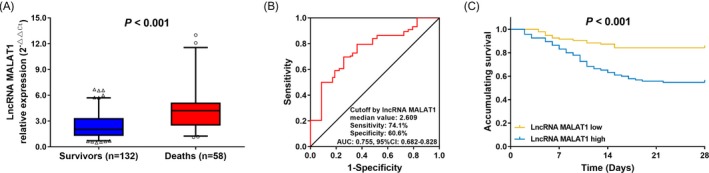
Expression of lncRNA MALAT1 in predicting 28‐day mortality in sepsis patients. The plasma expression of lncRNA MALAT1 in deaths and survivors (A), the ROC curve of lncRNA MALAT1 in predicting 28‐day mortality (B) and Kaplan‐Meier curves of patients with high and low lncRNA MALAT1 expressions (C). Comparisons were determined by Wilcoxon rank sum test. ROC curves were plotted, and AUC with 95% confidence interval was calculated to assess the discrimination ability of LncRNA MALAT1 for 28‐day mortality. Kaplan‐Meier curve was used to illustrate accumulating survival, and difference between two groups was determined by log‐rank test. *P* value < 0.05 was considered significant. MALAT1, metastasis‐associated lung adenocarcinoma transcript 1; ROC, receiver operating characteristic; AUC, area under the curve

The 28‐day mortality rate was 30.5%, and lncRNA MALA1 expression in plasma was elevated in deaths compared with survivors (*P* < 0.001) (Figure 2A), and then, the ROC curve analysis illuminated that lncRNA MALAT1 expression could predict the higher risk of 28‐day mortality with an AUC of 0.755 (95% CI: 0.682‐0.828; Figure 2B). Furthermore, according to median value of lncRNA MALAT1 expression in sepsis patients, patients were divided into those with lncRNA MALAT1 high expression and those with lncRNA MALAT1 low expression, and the accumulating survival was worse in patients with lncRNA MALAT1 high expression compared with that in patients who had lncRNA MALAT1 low expression (*P* < 0.001; Figure 2C). These data indicated that plasma lncRNA MALAT1 expression may serve as a prognostic biomarker in sepsis.

### 3.5 Correlation of plasma lncRNA MALAT1 with miR‐125b and implication of miR‐125b in sepsis

**Figure 3:**
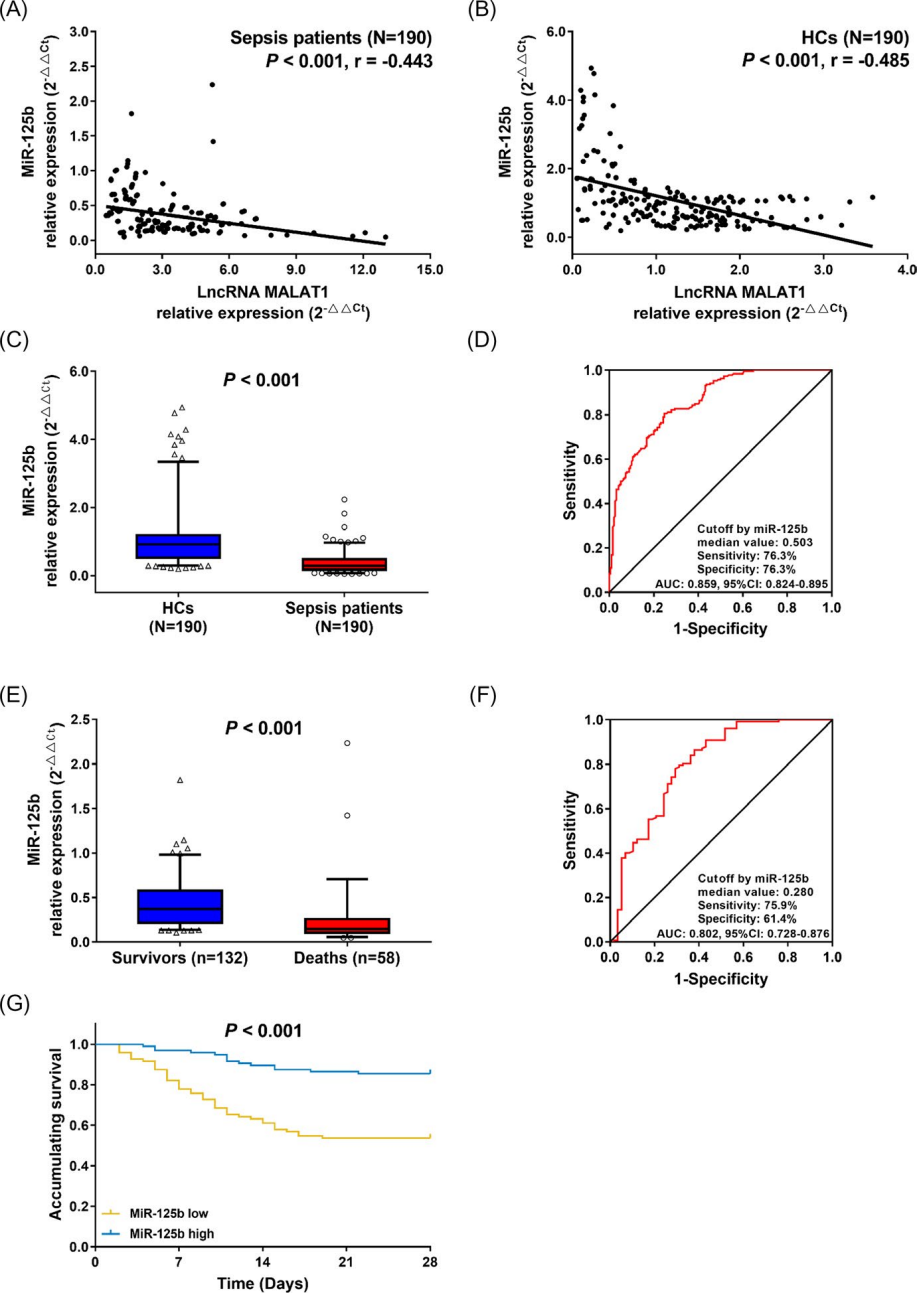
Correlation of lnRNA MALAT1 expression with miR‐125b. The correlation of plasma lncRNA MALAT1 expression with miR‐125b level in sepsis patients (A) and HCs (B), the expression of miR‐125b in sepsis patients and HCs (C), ROC curve of miR‐125b expression in predicting sepsis risk (D), miR‐125b expression in deaths and survivors (E), ROC curve of miR‐125b expression in predicting 28‐day mortality, (F) and the Kaplan‐Meier curves of patients with high and low miR‐125b expressions (G). Comparisons were determined by Wilcoxon rank sum test. ROC curves were plotted, and AUC with 95% confidence interval was calculated to assess the discrimination ability of miR‐125b for sepsis risk and 28‐day mortality. Kaplan‐Meier curve was used to illustrate accumulating survival, and difference between two groups was determined by log‐rank test. *P* value < 0.05 was considered significant. MALAT1, metastasis‐associated lung adenocarcinoma transcript 1; HCs, healthy controls; ROC, receiver operating characteristic; AUC, area under the curve

Moreover, we evaluated the correlation between lncRNA MALAT1 expression and miR‐125b expression in plasma, and found that lncRNA MALAT1 expression was negatively correlated with the miR‐125b expression in both sepsis patients (*P* < 0.001; Figure 3A) and HCs (*P* < 0.001; Figure 3B). Furthermore, miR‐125b expression was downregulated in sepsis patients compared with HCs (*P* < 0.001; Figure 3C), and ROC curve analysis showed that miR‐125b expression had a good value in differentiating sepsis patients from HCs with an AUC of 0.859 (95% CI: 0.824‐0.895; Figure 3D). In addition, miR‐125b level was decreased in deaths compared with survivors (*P* < 0.001; Figure 3E), and ROC curve analysis revealed that miR‐125b had a good predictive value for 28‐day mortality with an AUC of 0.802 (95% CI: 0.728‐0.876; Figure 3F). Moreover, patients with miR‐125b low expression presented with worse accumulating survival compared with patients who had miR‐125b high expression (*P* < 0.001; Figure 3G). In addition, with respect to all the continuous variables of clinical characteristics, miR‐125b expression was negatively correlated with SCr (*P* < 0.001), WBC (*P* = 0.007), CRP (*P* < 0.001), PCT (*P* < 0.001), TNF‐α (*P* < 0.001), IL‐6 (*P* < 0.001), IL‐8 (*P* < 0.001) and IL‐17 (*P* < 0.001) levels, and the APACHE II score (*P* < 0.001) as well as SOFA score (*P* < 0.001), while was not associated with age (*P* = 0.641), BMI (*P* = 0.659), or albumin level (*P* = 0.171) in sepsis patients (Table 4). As for the categorical variables, the miR‐125b expression was negatively correlated with history of cirrhosis (*P* < 0.001), but it was not correlated with gender (*P* = 0.506), smoke (*P* = 0.706), history of COPD (*P* = 0.133), history of cardiomyopathy (*P* = 0.174), or history of chronic kidney failure (*P* = 0.170) in sepsis patients (Table 5).

**Table 4:** Correlations of miR‐125b relative expression with clinical characteristics (continuous variables)

Items	MiR‐125b	
	*P* value	Correlation coefficient (*r*)
Age	0.641	−0.034
BMI	0.659	−0.032
Scr	<0.001	−0.265
Albumin	0.171	0.100
WBC	0.007	−0.194
CRP	<0.001	−0.374
PCT	<0.001	−0.260
APACHE II score	<0.001	−0.511
SOFA score	<0.001	−0.399
TNF‐α	<0.001	−0.418
IL‐6	<0.001	−0.339
IL‐8	<0.001	−0.324
IL‐17	<0.001	−0.418

Note

Correlation analyses were determined by Spearman's rank correlation test.

Abbreviations: APACHE, acute physiology and chronic health evaluation; BMI, body mass index; CRP, C‐reactive protein; IL, interleukin; PCT,, procalcitonin; Scr, serum creatinine; SOFA, sequential organ failure assessment; TNF, tumor necrosis factor; WBC, white blood cell.

**Table 5:** Correlations of miR‐125b relative expression with clinical characteristics (categorical variables)

Items	MiR‐125b, median (IQR)	*P* value
Gender		
Male	0.283 (0.171‐0.471)	0.506
Female	0.272 (0.146‐0.482)	
Smoke		
Yes	0.288 (0.128‐0.578)	0.706
No	0.273 (0.176‐0.434)	
History of COPD		
Yes	0.504 (0.188‐0.658)	0.133
No	0.272 (0.165‐0.427)	
History of cardiomyopathy		
Yes	0.239 (0.171‐0.619)	0.174
No	0.286 (0.144‐0.422)	
History of chronic kidney failure		
Yes	0.183 (0.063‐0.861)	0.170
No	0.286 (0.168‐0.471)	
History of cirrhosis		
Yes	0.471 (0.413‐0.640)	<0.001
No	0.240 (0.160‐0.394)	

Note

Correlation analyses were determined by Wilcoxon rank sum test.

Abbreviation: COPD, chronic obstructive pulmonary disease.

## 4 DISCUSSION

Sepsis, a relatively common and critically fatal SIRS in ICU, requires for immediate diagnosis and intervention for the enhancement of satisfactory clinical outcomes. However, the present biomarkers in clinical practice are not sensitive or specific enough for enhancing the timely intervention of sepsis, such as CRP, PCT, and IL‐6.^18^, ^19^, ^20^ Therefore, the effort for exploring biomarkers assisting in diagnosis and prognosis is continuing even at the time of writing, and increasingly profound genetic mechanisms of the interactions that take place in sepsis have been discovered, in which the roles of ncRNAs have been implied in the regulation of sepsis etiology.^21^ To be specific, lncRNAs have been revealed to play essential roles in the regulations of immune responses and inflammation in sepsis, indicating that they may hold missing drivers of the sepsis pathogenesis.^14^, ^22^ Herein, we investigated the correlation of a lncRNA that has a promising role in inflammation, lncRNA MALAT1, with the sepsis risk, and severity, inflammation level, and prognosis in sepsis patients, and found that: (a) plasma lncRNA MALAT1 was upregulated in sepsis patients and had good value in predicting sepsis risk; (b) lncRNA MALAT1 expression was positively correlated with severity and inflammation level in sepsis patients; (c) high lncRNA MALAT1 had good value in predicting increased 28‐day mortality and was associated with worse accumulating survival in sepsis patients; and (d) lncRNA MALAT1 expression was negatively correlated with miR‐125b expression in both sepsis patients and HCs.

LncRNA MALAT1 has been reported to participate in several inflammation‐related diseases in regulating the inflammatory or immune responses. An in vitro experiment shows that lncRNA MALAT1, mainly located in nuclear speckles, was upregulated in endothelial cells incubated with high glucose concentration and is correlated with higher expressions of inflammatory cytokines, and the diabetic‐induced inflammatory cytokines levels elevations are reduced in lncRNA MALAT1 knockdown diabetic rat models.^23^ Another in vitro experiment reports that lncRNA MALAT1 might play a pro‐inflammatory role in lung injury by regulating macrophage polarization via mediating the IL‐4 expression.^24^ And an in vitro and in vivo experiments elucidate that lncRNA MALAT1 advocates the inflammation‐mediated progression of hepatocellular carcinoma via binding to chromatin‐remodeling subunit BRG1.^14^ Recently, a study investigates the effect of lncRNA MALAT1 on septic lung injury and finds that lncRNA MALAT1 knockdown markedly reduced lung injury induced by sepsis possibly via the repression of p38 MAPK/p65 NF‐κB signaling pathway.^25^ Another experiment illuminates that lncMALAT1 knockdown helps with the suppression of inflammatory response via elevating the miR‐146a in lipopolysaccharide‐induced acute lung damage.^26^ These previous studies indicate that lncRNA MALAT1 acts as a pro‐inflammatory gene and also promotes organ injury, such as the heart and lung injuries. In addition, there are reports illuminating that lncRNA MALAT1 reduces inflammation in several diseases as well. For instance, a recent experiment reveals that lncRNA MALAT1 represses proliferation and inflammation by inhibiting Wnt signaling pathway in fibroblast‐like synoviocytes.^15^ And another study elucidates that lncRNA MALAT1 expression is elevated in ischemia‐reperfusion injury and represses hypoxia‐induced inflammation via the NF‐κB pathway.^27^ And these findings indicate a dual role of this lncRNA in the regulation of inflammation probably because of that in lncRNA MALAT1 might function as a gene that keep balance in inflammatory responses, while in sepsis the main pathological feature is an inflammation cascade, which results in a pro‐inflammatory role of lncRNA MALAT1 in the complex interactions of sepsis. In this study, we discovered that upregulated plasma lncRNA MALAT1 expression correlated with higher sepsis risk, and worse disease condition, increased inflammation level, and poorer prognosis in sepsis patients, which could be explained by that lncRNA MALAT1 might promote the development and progression of sepsis through (a) enhancing inflammation via mediating multiple signaling pathways or proteins, such as regulating the IL‐4 expression and (b) advocating the organ dysfunction by regulating various pathways, for instance the p38 MAPK/p65 NF‐κB signaling pathway.^14^, ^23^, ^24^, ^25^

In addition, we evaluated the expression of lncRNA MALAT1 in plasma in sepsis patients, and the possible sources of lncRNA MALAT1 in plasma might be exosomes or cell debris from inflammatory cells or immune cells such as lymphocytes and macrophages due to that most lncRNAs in plasma are abundantly expressed in exosomes or cell debris or exist as nucleic acid, and sepsis is a type of systemic inflammatory response syndrome closely related to inflammation and immune disorders.^28^, ^29^

MiR‐125b is an inflammatory‐related miRNA and has been reported to play an anti‐inflammatory role in various diseases. For example, a cells experiment reveals that miR‐125b suppresses lipopolysaccharide‐induced inflammatory damage in chondrogenic cells ATDC5 by targeting macrophage inflammatory protein‐1 alpha (MIP‐1α).^30^ And another study elucidates that miR‐125b promotes the recovery and regeneration of spinal cord injury via decreasing cell apoptosis and inflammatory responses in neurons obtained from mouse models of cervical spinal cord contusion.^31^ In addition, there are several previous experiments illustrating that lncRNA MALAT1 targets miR‐125b to regulated inflammation or other pathogenesis progresses in several diseases. For instance, an experiment elucidates that lncRNA MALAT1 promotes neovascularization via mediating the miR‐125b/VE‐cadherin axis in endothelial cells models of diabetic retinopathy.^32^ More importantly, a study illuminates that lncRNA MALAT1 promotes cardiac inflammation and dysfunction in sepsis mice by mediating miR‐125b and p38 MAPK/NFκB.^17^ In our study, the plasma expression of lncRNA MALAT1 was negatively associated with miR‐125b expression in both sepsis patients and HCs, and further analysis showed that miR‐125b level was downregulated in sepsis patients and nonsurvivors, and its downregulation was correlated with increased severity, inflammation level, and worse survival in sepsis patients, indicating that miR‐125b is a target of lncRNA MALAT1 in sepsis and plays an anti‐inflammatory role in sepsis.

There were several limitations in our study. First, our study was conducted in a single center, which caused a selection bias due to that the patients were mostly from the same region in China. Second, the molecular mechanisms of lncRNA MALAT1 in promoting the development or progression of sepsis was not evaluated in our study. Third, long‐term clinical outcomes were not assessed in this study. Thus, multicenter studies with longer follow‐up time, and in vivo as well as in vitro experiments, should be performed in the future.

In conclusion, lncRNA MALAT1 could be developed as a biomarker for facilitating diagnosis and management in sepsis patients.

## CONFLICT OF INTEREST

The authors declare that they have no conflicts of interest.
